# An explainable deep learning framework for few shot crop disease detection in rice and sugarcane using CNN based feature extraction

**DOI:** 10.1038/s41598-026-37501-2

**Published:** 2026-03-05

**Authors:** Heba El-Behery, Abdel-Fattah Attia, Nermeen Gamal Rezk

**Affiliations:** 1 Department of Computer and Systems Engineering, Faculty of Engineering, Kafrelsheikh University, Kafr_El_Sheikh, 6860404 Egypt; 2 Intelligent System Research Group (ISRG), Kafrelsheikh University, Kafr el-Sheikh, Egypt

**Keywords:** Smart farming, Explainable artificial intelligence (XAI), Prototypical networks and model-agnostic meta-learning (MAML), Grad-CAM explanation, Computational biology and bioinformatics, Mathematics and computing, Plant sciences

## Abstract

Where crop health is essential to global food security. Our focus is on early crop disease detection in the field of agriculture, especially Rice and Sugar cane leaf disease. This prompts researchers to consider quick, automated, cost-effective, precise, and efficient methods of identifying the kinds of diseases utilizing contemporary technologies like image processing, artificial intelligence (AI), and Explainable Artificial Intelligence (XAI). This paper proposes an framework to detect pest infestation for rice and Sugar cane cultivation and suggests an effective framework for rice and Sugar cane disease detection and forecasting that uses image processing to standard, resizing, and normalization rice and Sugar cane images then, using feature extractor using CNN after that we using few-shot learning (FSL) techniques such as like Prototypical Networks and Model-Agnostic Meta-Learning (MAML) learning techniques for superior decision-making in smart farming systems. The experimental findings demonstrated the Accuracy and specificity of the suggested framework in identifying and effectively predicting the kind of disease. According to the results, the suggested framework outperformed the state-of-the-art benchmark algorithms in disease prediction while producing results that were plausible. With Prototypical Networks and MAML for rice leaf disease datasets, it increased by up to 97.6% and 95.27%, respectively. For effective rice disease identification, Prototypical Networks and MAML for Sugar cane leaf disease datasets increased by up to 91.68% and 90.27%, respectively. Interpretable AI-driven insights were further made possible by the combination of proposed system with Grad-CAM Explanation, which improved decision-making transparency.

## Introduction

Global food production is seriously threatened by plant diseases, particularly in economies that rely heavily on agriculture^1^. Certain diseases can yield losses in crops like rice and sugar cane^4^. Conventional manual detection is time-consuming and needs knowledge that remote farmers frequently lack^6^. Developments in deep learning and artificial intelligence (AI) enable automatic disease categorization from leaf photos^3^, enabling more rapid, economical, and real-time field responses^2^. Large datasets, like the Sugar cane Leaf Disease Dataset^8^ and various rice disease datasets^9^ are used to train AI algorithms that efficiently identify and classify diseases early on, encouraging prompt responses and lowering the use of pesticides. Through precise disease control solutions for rice and sugarcane farming^5^, the improvement of dataset quality and model generalization seeks to maximize yields and promote sustainable agriculture.

Improving dataset quality and model generalization under various environmental conditions are the main goals of this field of study. AI can optimize yield projections, reduce crop losses, and advance sustainable agriculture by utilizing these datasets^7^. This work focuses on the identification and classification of illnesses that affect rice and sugarcane, two significant crops, using AI, particularly with the use of specialized image datasets. The availability of comprehensive and well-chosen datasets is crucial for the development and evaluation of trustworthy AI-powered disease detection systems. This introduction emphasizes the importance of these datasets in furthering AI research for agricultural applications, particularly focusing on the Sugar cane Leaf Disease Dataset and other rice datasets. With the help of these datasets, sophisticated machine learning models may be trained, leading to innovative solutions that could revolutionize the management of diseases in rice and Sugar cane cultivation.

This paper’s primary contributions are:

- This process provides useful information regarding the primary causes of Rice and Sugar cane disease and enhances the model’s functionality. VGG19, RES50, VGG16, XCEPTION, Protypical network, and MAML were used for modeling. To explain why crops are likely to develop disease or not, a few-shot learning approaches, like Prototypical Networks and Model-Agnostic Meta-Learning, were also employed and combined with the XAI.
- A suggested preprocessing methodology, that uses image processing to standard, resizing, and normalizing rice and Sugar cane images then, using feature extractor using CNN), has been put into practice to address missing values in datasets, producing better results by incorporating tried-and-true methods in contrast to current approaches. Combining FSL and XAI techniques enables precise disease prediction and comprehensible explanation.
- By comparing our suggested model’s performance to that of state-of-the-art models that had previously been applied to the same dataset, we were able to demonstrate its superior performance in terms of Accuracy, precision, recall, score, ROC curve, interpretability, and resilience.
- Because of its scalability and adaptability, the suggested model is especially made for smart farming applications, improving the intelligent prediction and monitoring of disease crops.

## Paper organization

The remainder of this paper is structured as follows: “Related work” presents a detailed review of related works, discussing previous studies on identification and classification of diseases that affect rice and sugarcane. “Methodology” describes the proposed methodology, including the framework. “Experimental results” provides experimental results and analysis, comparing the proposed with traditional methods. Finally, “Conclusion and future scope” concludes the paper with key findings and future research directions.

## Related work

A new Generative Adversarial Network (GAN) model called SugarcaneGAN is presented by the authors in^10^. It is intended to efficiently expand the sample of crop disease traits, especially for striped sugarcane leaves under complex backgrounds. To get around the limitations of earlier GANs (such as LeafGAN and STA-GAN) that used Grad-CAM, SugarcaneGAN uses a lightweight U-RSwinT module that combines the advantages of CNN and Swin Transformer to produce high-quality lesions and extract leaves accurately.

In^11^ authors introduce first extract features from photos of rice leaf damage using convolution neural networks (CNNs), the specific disease is then classified and predicted using the SVM approach. The experimental findings demonstrate that the deep learning and SVM-based rice diseases detection model has an average correct recognition rate of 96.8%. A CNN model called InceptionResNetV2 is used in conjunction with a transfer learning technique to identify diseases in images of rice leaves and achieved a respectable Accuracy of 95.67%^12^. By using Deep Learning (DL) and transferring learning approaches, the authors in^12^ compiled a comprehensive dataset of 5932 self-generated photos of rice leaves and reported an astonishing Accuracy of 99.94% with a customized VGG16 model.

The authors in^13^ suggest a deep learning method for rice leaf disease detection on mobile devices using EfficientNet with transfer learning, achieving 95% validation Accuracy and deploying it as an Android application. The authors in^14^ use the deep learning model with four stages (pre-processing, feature extraction, feature selection, and classification) and an adaptive bi-long short-term memory (ABi-LSTM) classifier for paddy leaf diseases.

The authors in^16^ suggest a deep learning neural network architecture that uses images of diseased leaves to forecast the type of disease affecting the sugarcane crop, reporting 96% Accuracy. To improve sugarcane disease prediction, the authors in^18^ present effective comparison of deep learning models (AlexNet, ResNet18, VGG19, DenseNet201) and reported VGG19 achieving 98.82% Accuracy. The authors in^19^ investigate the use of deep learning for sugarcane leaf disease classification using ResNet-50, VGG-16, DenseNet-201, VGG-19, and Inception V3 on 2511 images, with ResNet-50 achieving the best performance of 95.69% Accuracy.

The authors in^20^ study assesses how activation functions affect the AlexNet deep learning model’s ability to classify diseases in sugarcane, with LeakyReLU achieving 90.67% Accuracy. With a focus on three categories (healthy, rusty, and yellow leaf disease), the authors in^21^ investigated CNN versus traditional methods and achieved 97% Accuracy. In^22^ they use EfficientNet with transfer learning and fine-tuning, achieving 94.60% Accuracy on sugarcane leaf diseases. In^23^ the authors present a novel adaptation of the learning rate decay policy with MobileNet-V2 as backbone on a small-scale sugarcane dataset. In^24^ the authors suggested framework builds a prediction model using AutoML technology with explainable AI (XAI) techniques for paddy crop diseases.

To fill these gaps, this study presents a unique method for detecting diseases in Rice and Sugar cane crops that combine interpretability techniques like Grad-CAM with Explainable FSL models, namely Prototypical Network and MAML. Explainable FSL, in contrast to conventional approaches, achieves great Accuracy even with limited real-world data and offers interpretable insights into the decision-making process. Additionally, this study closes a significant gap in literature and advances the development of effective, precise, and transparent disease detection systems for agriculture by showcasing the better performance and interpretability of Explainable FSL models.

## Methodology

**Fig. 1:**
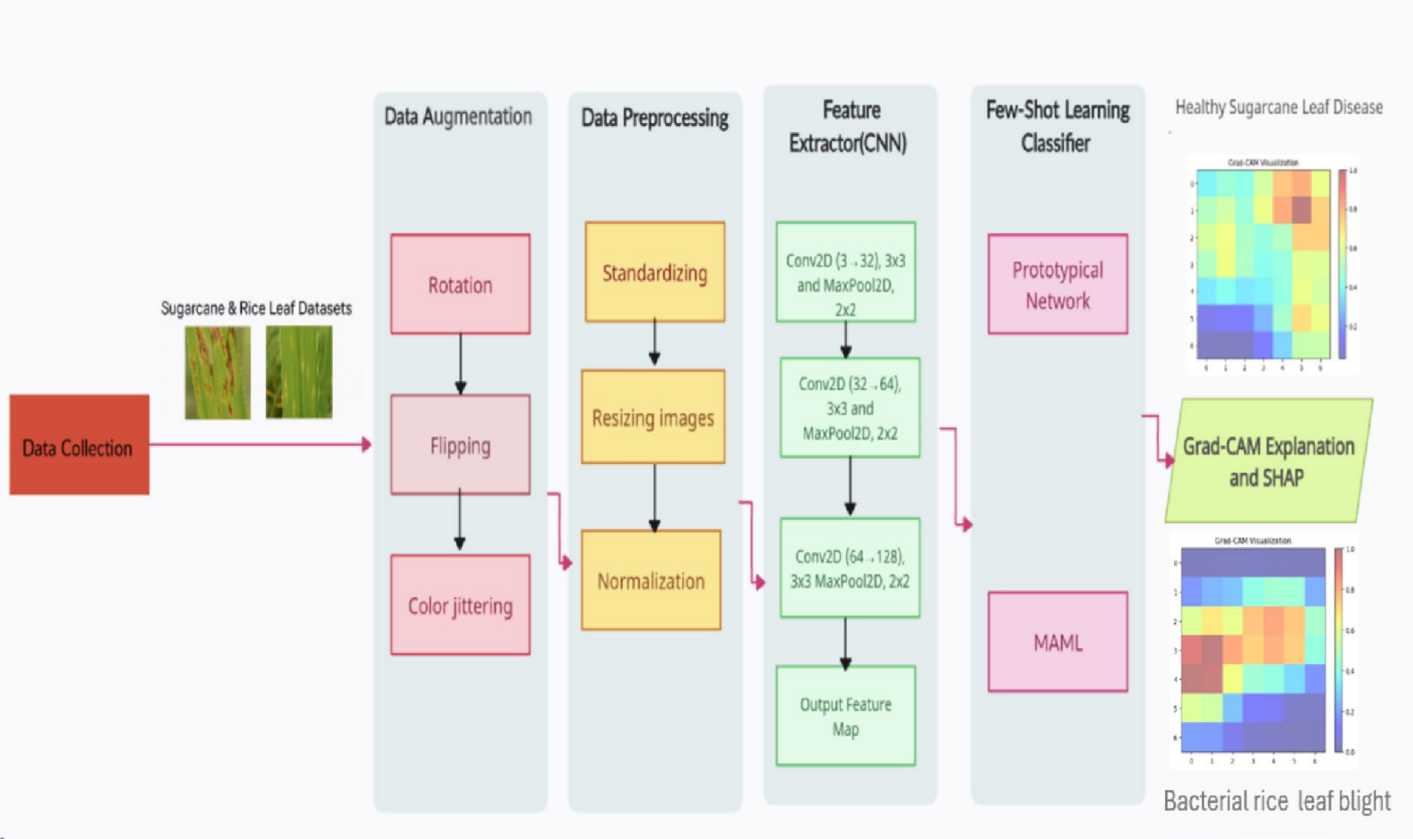
Framework for proposed system.

In this section, we now present our proposed framework that specifically addresses the identified gaps in few-shot capability and interpretability. As seen in Fig. 1, which explains the use of important AI approaches for disease identification in rice and sugar cane production, this framework especially considers two publicly available datasets: the Sugar cane Leaf Disease Dataset and the UCI Rice Leaf Diseases dataset. These techniques include data collection, data augmentation, data preprocessing, few-shot learning, and XAI explanation.

### Data collection

For this framework, we will primarily utilize the two provided datasets as shown in Table 1, first dataset is Sugarcane Leaf Disease collection which is a manually gathered image collection that focuses exclusively on illnesses that afflict Sugar cane leaves. It is recognized by the DOI 10.17632/9424skmnrk.1^8^ and is accessible via Mendeley Data.49 The 2569 photos in this dataset are divided into five main categories: mosaic, redrot, rust, yellow disease, and healthy.31 In order to intentionally create variance in terms of image resolution and quality and to better portray the variability found in the actual world, the images were taken with smartphones that had different configurations. Every photograph in the collection is saved as a JPG file in the RGB color format. The dataset’s image sizes vary because different smartphone cameras were used to collect data.

The second dataset is UCI Rice Leaf Diseases dataset, is a smaller but still useful dataset, featuring 120 photos distributed equally across three classes: Bacterial leaf blight, Brown spot, and Leaf smut. In contrast to datasets gathered in the field, the images in this dataset were taken in a more controlled setting that is, with a white background and direct sunshine^9^ .

**Table 1:** Characteristics of the rice and sugarcane leaf disease datasets.

Dataset	Source and link/DOI	Images	Classes (count)	Image conditions	License
Sugarcane leaf disease dataset	Mendeley Data, DOI: 10.17632/9424skmnrk.1	2569	Healthy (511), Mosaic (498), Red Rot (520), Rust (491), Yellow (549)	Real-field, various phones	CC BY 4.0
UCI rice leaf diseases dataset	UCI machine learning repository	120	Bacterial leaf blight (40), Brown spot (40), Leaf smut (40)	Controlled white background	Public

### Data augmentation techniques for plant disease images

The available image datasets must frequently be supplemented using a variety of methods to create reliable and generalizable models for plant disease diagnosis^25^. Geometric transformations entail adjusting the images’ spatial characteristics, including flipping and rotation^26^. Through the creation of altered replicas of the original photos, these changes artificially expand the training data’s size and diversity. Without adding new visual information, the dataset can be further expanded by flipping photographs either vertically or horizontally^27^. The model’s capacity to generalize to real-world situations where such modifications are frequent is enhanced by training on these altered images, which makes it more resistant to changes in the location and orientation of the diseased plant components.

An further set of methods for enhancing data is provided by color space modifications. This entail introducing color jitter or noise and modifying the images’ brightness, contrast, saturation, and other color characteristics^28^. Different lighting conditions that can be experienced in the field can be simulated by adjusting the contrast, brightness, or color balance. To make the model less sensitive to small changes in image quality, apply blur or sharpening effects. To further increase the variety of the dataset, color jittering entails making tiny, random adjustments to the pixel color values^29^. Training on photos that include these color space adjustments makes the model more robust to changes in lighting and general image quality, which are frequent problems in actual agricultural environments.

### Data preprocessing steps

Before image data can be effectively utilized for training models that detect plant diseases, several essential preprocessing steps are typically necessary. Image resizing is a fundamental step that involves adjusting the dimensions of all images in the dataset to a uniform size. This consistency is particularly important for deep learning models like Convolutional Neural Networks (CNNs)^30^, which often require fixed-size input images. Common resizing dimensions include 256 × 256 or 224 × 224 pixels. Normalization is another critical preprocessing step that standardizes the pixel values across the dataset, often by scaling them to a specific range, such as 0 to 1. This process helps manage variations in contrast and lighting conditions across different images, which can accelerate the training process and enhance the model’s stability. These preprocessing steps ensure that the image data is in a suitable format for machine learning models, leading to more efficient training and potentially higher Accuracy in disease detection^31^.

### Few-shot learning in plant disease identification

In the field of plant disease identification, collecting large, labeled datasets for every potential disease and growth stage can be a significant challenge. This is where few-shot learning (FSL) approaches become particularly relevant, as they enable image classification even when only a minimal number of labeled examples are available for each disease category^32^. Several approaches exist within few-shot learning, each with its approach to tackling the data paucity problem.

FSL strives to construct models that can generalize effectively to novel categories or tasks using only a minimal number of labeled samples. Among the various FSL approaches, MAML and Prototypical Networks have garnered significant attention for their effectiveness in image classification tasks with limited data^33^. MAML is a meta-learning technique that learns a decent initialization of model parameters, enabling quick adaptation to new tasks with only a few gradient steps and limited data. Conversely, Prototypical Networks are a metric-based FSL technique that learns an embedding space in which a prototype represents each class, and classification is carried out by evaluating how close fresh cases are to these prototypes^34^.

The goal of MAML, a meta-learning algorithm, is to train a model’s initial parameters so that it can efficiently adapt to a new task sampled from a distribution of tasks using only a few data points and training iterations. The main idea behind MAML is to find a parameter initialization that is sensitive to changes across different tasks, hence facilitating rapid adaptation. The algorithm works by training the model to be easily fine-tuned for new, undetected tasks^36^.

A batch of tasks is sampled at every meta-training iteration. The model uses a limited support set of examples to assess the gradient of the task-specific loss function with respect to the present parameters for each task. After that, it uses a task-specific learning rate to update the starting parameters through one or more gradient descent stages to compute the modified parameters. Then, using a meta-learning rate, the initial parameters are modified according to the total of the losses on the query sets of the modified models for every task that was sampled. Model and task agnosticism are important features of MAML^35^. It does not enforce specific limitations on the model architecture and can be used with any model trainable via gradient descent, including convolutional neural networks (CNNs).

For few-shot learning, MAML is a well-liked and successful meta-learner in a variety of fields. Continuous research aims to improve MAML’s performance, especially in terms of generalization and Accuracy, even though it offers a good foundation for tackling the problem of sparse data in plant disease identification. Developing better meta-learning techniques or improving the application of MAML to problem characteristics in plant pathology are frequently the main goals of these initiatives.

MAML has been extended and deployed for image-based plant disease classification applications, frequently exploiting the capabilities of recent deep learning architectures as base learners^37^.

Prototypical Networks provide an alternative to few-shot learning by concentrating on learning a metric space where classification can be carried out based on the distance to prototype representations of each class. The basic idea is that data points from the same class tend to cluster around a single prototype within the learned embedding space. The algorithm entails first learning an embedding function, usually a neural network, that maps input data into a lower-dimensional feature space^38^. A prototype vector is then calculated as the mean of the embedded support points for each class in the support set (a small set of labeled examples for new classes). Generally, Prototypical Networks are trained in an episodic fashion, with each training episode simulating the few-shot learning task, with a randomly chosen subset of classes as the support set and a few examples as the query points for each class, where the model learns by minimizing the negative log-probability of the true class for the query points based on their distances to the prototypes calculated from the episode’s support set.

An embedding network, usually a convolutional neural network (CNN), trained to extract meaningful visual features from the plant images, generates the feature embeddings. In Prototypical Networks, each plant disease class is represented by a prototype vector, which is determined by taking the meaning of the feature embeddings of the support set images that belong to that specific disease class^39^. Prototypical Networks are particularly well-suited for learning a metric space for classification with limited data, making them relevant for plant disease identification, where acquiring large, labeled datasets can be difficult.

After establishing prototypes for each disease class in the support set, new unseen plant images (query instances) are classified by first embedding them into the same feature space using the same embedding network, then calculating the distance between the query image’s embedding and each of the class prototypes using a distance metric, like Euclidean distance, and assigning the disease class corresponding to the prototype that is closest to the query image’s embedding in the feature space as the predicted disease for the new instance^40^. Prototypical Networks are computationally efficient and relatively simple to implement due to the ease of averaging embeddings to create prototypes. However, the effectiveness of these networks is largely reliant on the caliber of feature embeddings that the embedding network learns; if the embedding network is unable to identify the key characteristics that differentiate various plant diseases, the prototypes might not be representative, which could result in low classification Accuracy^41^.

### Explainable AI (XAI) for interpreting disease detection models

Several explainable AI (XAI) techniques are particularly applicable to the field of plant disease detection^42^, such as Grad-CAM (Gradient-weighted Class Activation Mapping) and SHAP (SHapley Additive exPlanations), which create heatmaps that overlay on the original image, meaning the areas that most significantly influenced the model’s classification decision. While having high Accuracy in plant disease detection is important, knowing why a model makes a particular prediction is equally important for fostering trust and facilitating informed decision-making^43^.

We can obtain important insights into the characteristics and patterns that the model learns to recognize when identifying illnesses in rice and Sugar cane by utilizing these XAI techniques. For example, XAI techniques can show whether a model focuses on the distinctive brown lesions with a yellow halo, which are important visual indications of this disease, if it correctly detects brown spot in a rice leaf^44^. Agricultural experts may verify the model’s behavior and make sure it is using visually sound, scientific criteria by comprehending these taught features. Because it builds trust and makes it possible for professionals to use these cutting-edge technologies for better crop health management, interpretability is essential for the practical adoption of AI-driven disease detection systems in agriculture.

Although there has been a lot of study on the individual components of data collection, augmentation, preprocessing, few-shot learning, and XAI in relation to plant disease diagnosis, there aren’t many studies that combine all these components into a single, comprehensive framework, especially for rice and sugarcane. The system should include few short learning approaches because it may be difficult to gather big, labeled datasets for all illnesses impacting rice and Sugar cane databases, especially in their early phases of development. XAI techniques ought to be a key component of the framework to guarantee openness and foster confidence in the system’s forecasts. Heatmaps created by Grad-CAM are superimposed on the original image to show the regions that influenced the model’s classification choice the most^45^.

A critical layer of interpretability is added to machine learning models for plant disease diagnosis by SHAP explanation. SHAP improves our comprehension of the model’s logic, helps us recognize important illness indicators, makes model improvement easier, and eventually promotes increased confidence and use of these potent diagnostic tools in agriculture by exposing the visual characteristics that inform the model’s predictions. Research on plant pathology that incorporates SHAP analysis provides important insights into the behavior of the model as well as the visual traits of plant diseases^46^.

## Experimental results

To train and evaluate the efficiency of our Proposed System, we use a widely known UCI Rice Leaf Diseases dataset and The Sugar cane Leaf Disease Dataset that are detailed in the data gathering step. The dataset is divided into two halves with an 80–20% training-test split. Standard statistical measures that are widely employed in machine learning classification, including recall, Accuracy, precision, F1-score, and ROC curve, were utilized to assess the performance of the suggested model. References^47^ contain the formulas for these metrics.

We used a 5-way 5-shot classification assignment with a query size of 10 samples per class to test our method under a typical few-shot learning paradigm. A new support and query set were randomly sampled throughout each of the 100 episodic training sessions for the model. The Adam optimizer was used to optimize the model with a CrossEntropy loss and a fixed learning rate of 1e-4. Class prototypes for the Prototypical Network were calculated as the mean of the support set embeddings, and the logits for the softmax function were obtained by calculating the negative squared Euclidean distance between query samples and these prototypes.

These measures are commonly used in practical machine learning research and are well known. As a gauge of the model’s overall correctness, Accuracy shows what percentage of the test set the classifier properly classifies. Recall assesses the completeness of correctly identified positive class examples, whereas precision gauges the Accuracy of positively labeled instances^48^. When combined, these indicators provide a thorough evaluation of a classification model’s performance. A workstation with an NVIDIA GeForce RTX 4060 Laptop GPU, was used for testing. The implementation was done using Python 3.8, and all calculations were done in interactive mode.

### Classification models performance

**Fig. 2:**
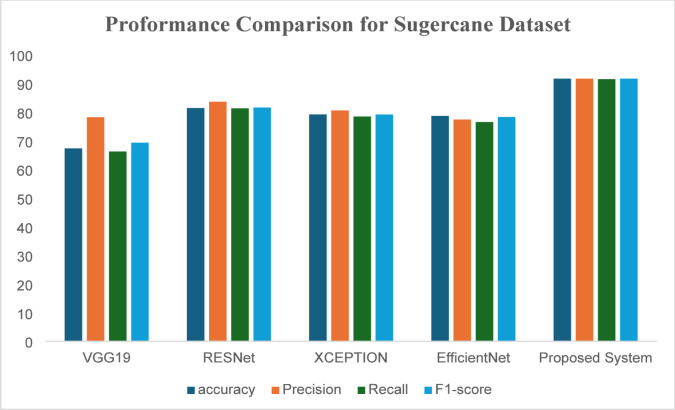
Comparison between our proposed system and recent methods according to sugar cane leaf dataset.

When applying the proposed model to the two datasets, use standard statistical measures and benchmark models like VGG19, ResNet, Xception, and EfficientNet, then implement a few-shot learning approaches to enhance the Accuracy of the plant disease classification model (Fig. 2).

**Table 2:** Different deep learning techniques result according to accuracy, recall, precision, and F1-score for proposed system using sugarcane leaf dataset.

Method	Accuracy	Precision	Recall	F1-score
VGG19	67.33	78.23	66.27	69.34
RESNet	81.42	83.64	81.33	81.61
XCEPTION	79.21	80.6	78.46	79.15
EfficientNet	78.66	77.43	76.56	78.31
MAML	85.6	86.01	85.5	85.1
Proposed system	91.68	91.68	91.50	91.68

**Fig. 3:**
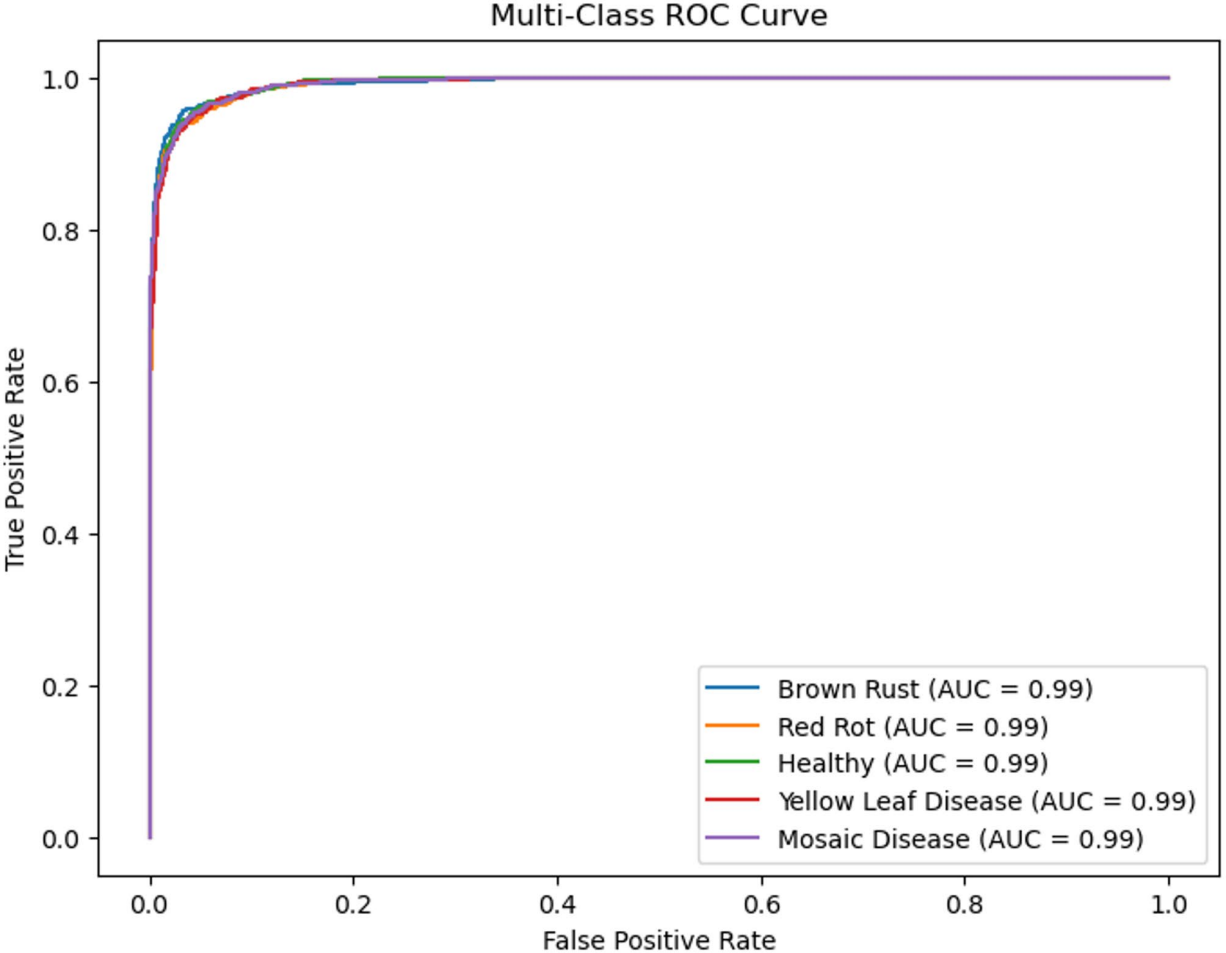
ROC curve along with the AUC values for proposed system using sugar cane leaf dataset.

**Fig. 4:**
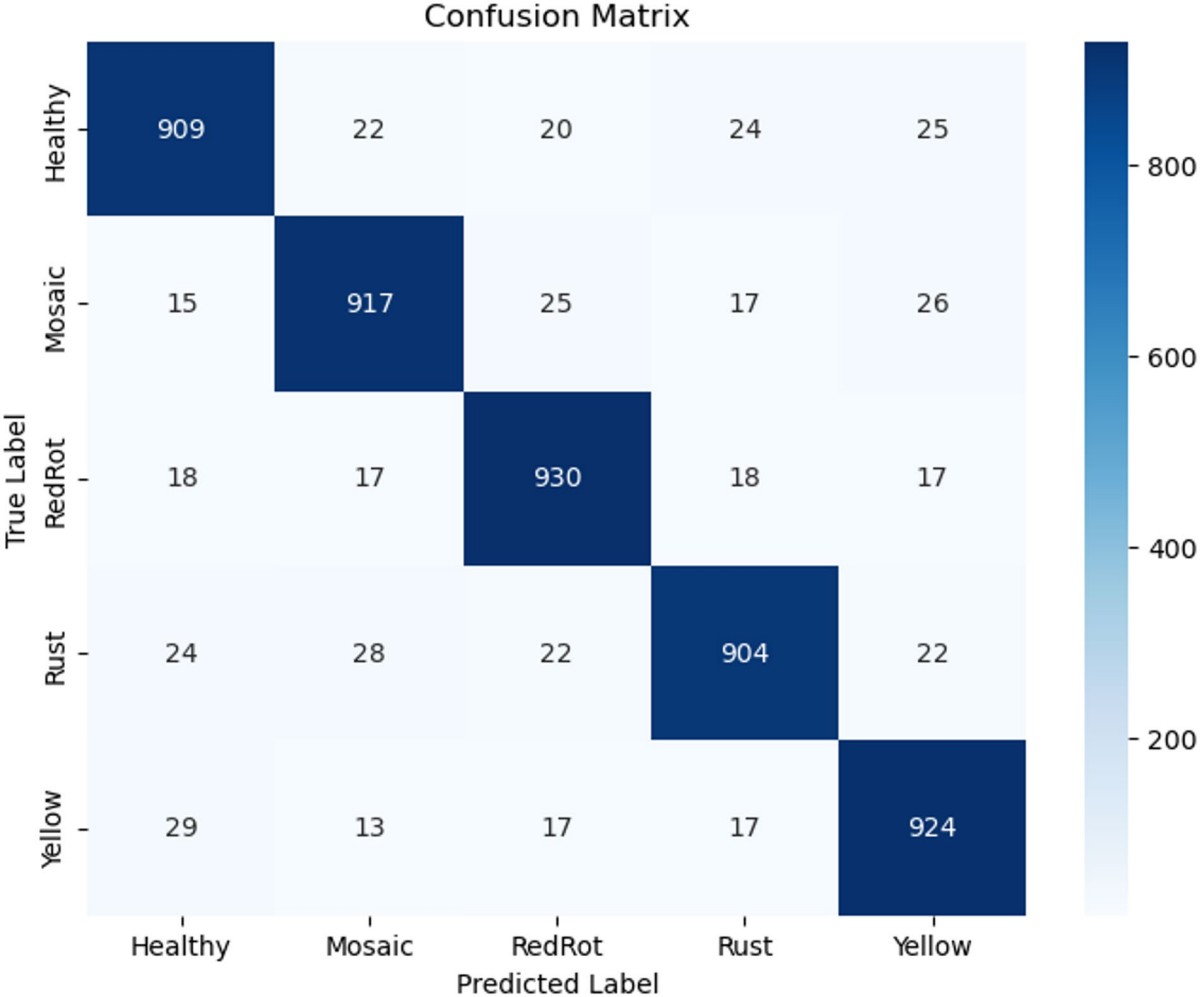
Confusion matrices for proposed system using sugar cane leaf dataset.

**Fig. 5:**
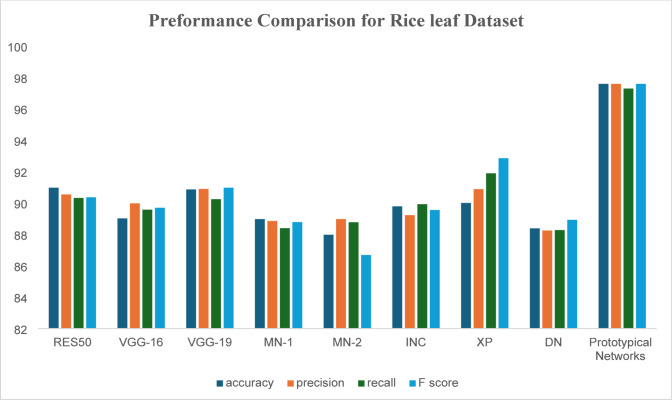
comparison between our proposed system and recent methods, according to rice leaf dataset.

The performance of these models is given in Table 2. Proposed System achieves the best performance compared to the other deep learning architectures due to its deep interpretations and effective handling of vanishing gradients. Despite this, the proposed Prototypical Networks model performs better than the baseline architecture. Figure 3 shows the receiver operating curve, which is commonly used in classification. It plots the true positive rate versus the false positive rate of the classifier. It is known that a perfect classifier will have ROC-area under a curve equal to 1, whereas a random classifier will have AUC equal to 0.5. It is evident from Fig. 3, AUC values are near 1, indicating proposed Prototypical Networks model is a good classifier. A confusion matrix is given in Fig. 4 for the proposed Prototypical Networks model classifier. Most of the images are on the principal diagonal which shows classification with the model is good (Fig. 5).

**Table 3:** Different deep learning techniques result according to accuracy, recall, precision, and F-score for proposed system using rice leaf dataset.

Algorithms	Accuracy	Precision	Recall	F-score
RES50	90.98	90.55	90.33	90.37
VGG-16	89.02	89.98	89.58	89.70
VGG-19	90.87	90.90	90.25	90.98
MN-1	88.98	88.86	88.40	88.79
MN-2	87.98	88.98	88.78	86.69
INC	89.79	89.23	89.93	89.56
XP	90.01	90.89	91.90	92.86
DN	88.39	88.25	88.28	88.93
Proposed system	97.6	97.6	97.3	97.6

**Fig. 6:**
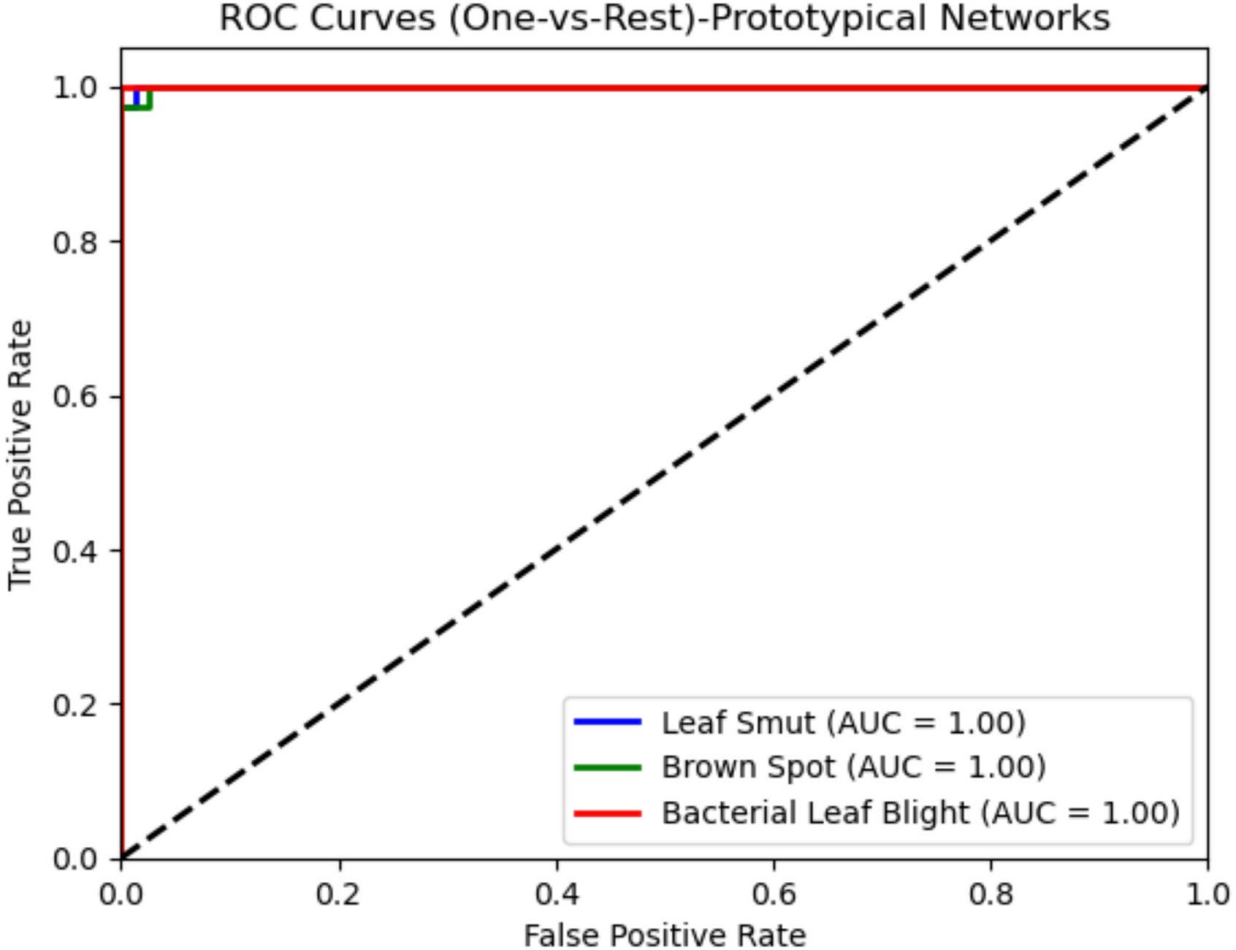
ROC curve along with the AUC values for proposed system using rice leaf dataset.

**Fig. 7:**
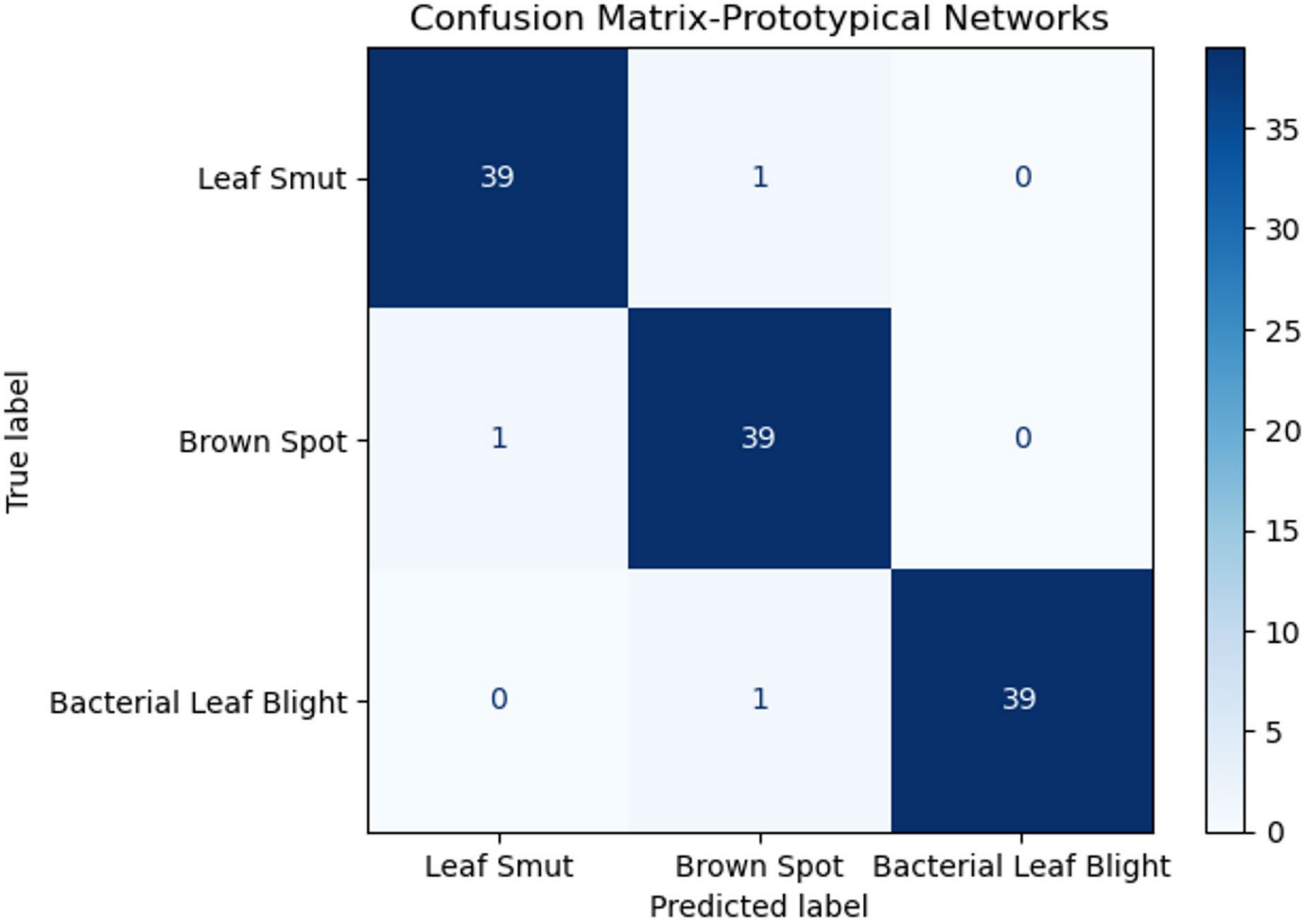
Confusion matrices for proposed system using Rice leaf dataset.

The performance of these models is given in Table 3. Proposed System achieves the best performance compared to the other deep learning architectures due to its deep interpretations and effective handling of vanishing gradients. Despite this, the proposed Prototypical Networks model performs better than the baseline architecture. A ROC Curve is given in Fig. 6 for the proposed Prototypical Networks model classifier. The results demonstrate exceptional diagnostic performance, with a perfect Area Under the Curve (AUC) of 1.00 achieved for all three classes: Leaf Smut, Brown Spot, and Bacterial Leaf Blight. Most of the images are on the principal diagonal, which shows classification with the model is good. A confusion matrix is given in Fig. 7 for the proposed Prototypical Networks model classifier. Most of the images are on the principal diagonal which shows classification with the model is good.

### XAI explanation

#### XAI sugar cane leaf disease dataset

**Fig. 8:**
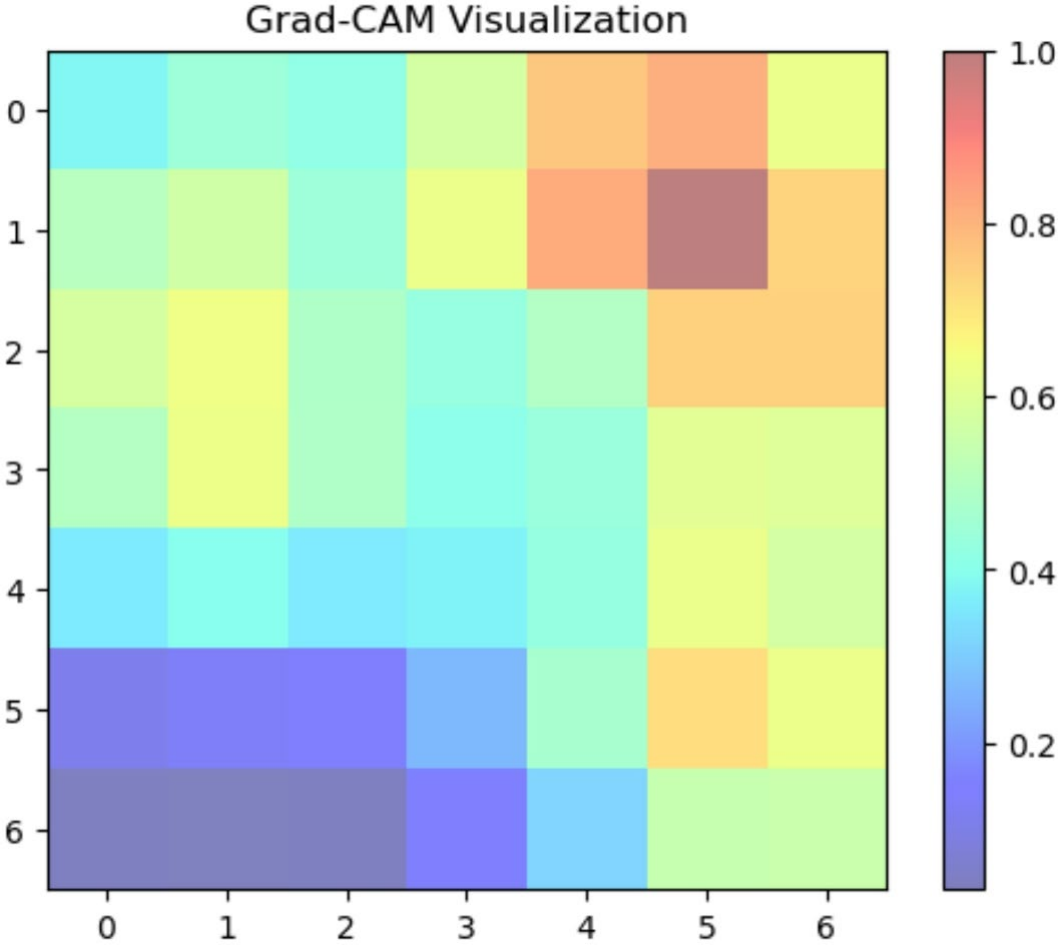
Grad-CAM activation map indicating healthy pattern influence on classification.

**Fig. 9:**
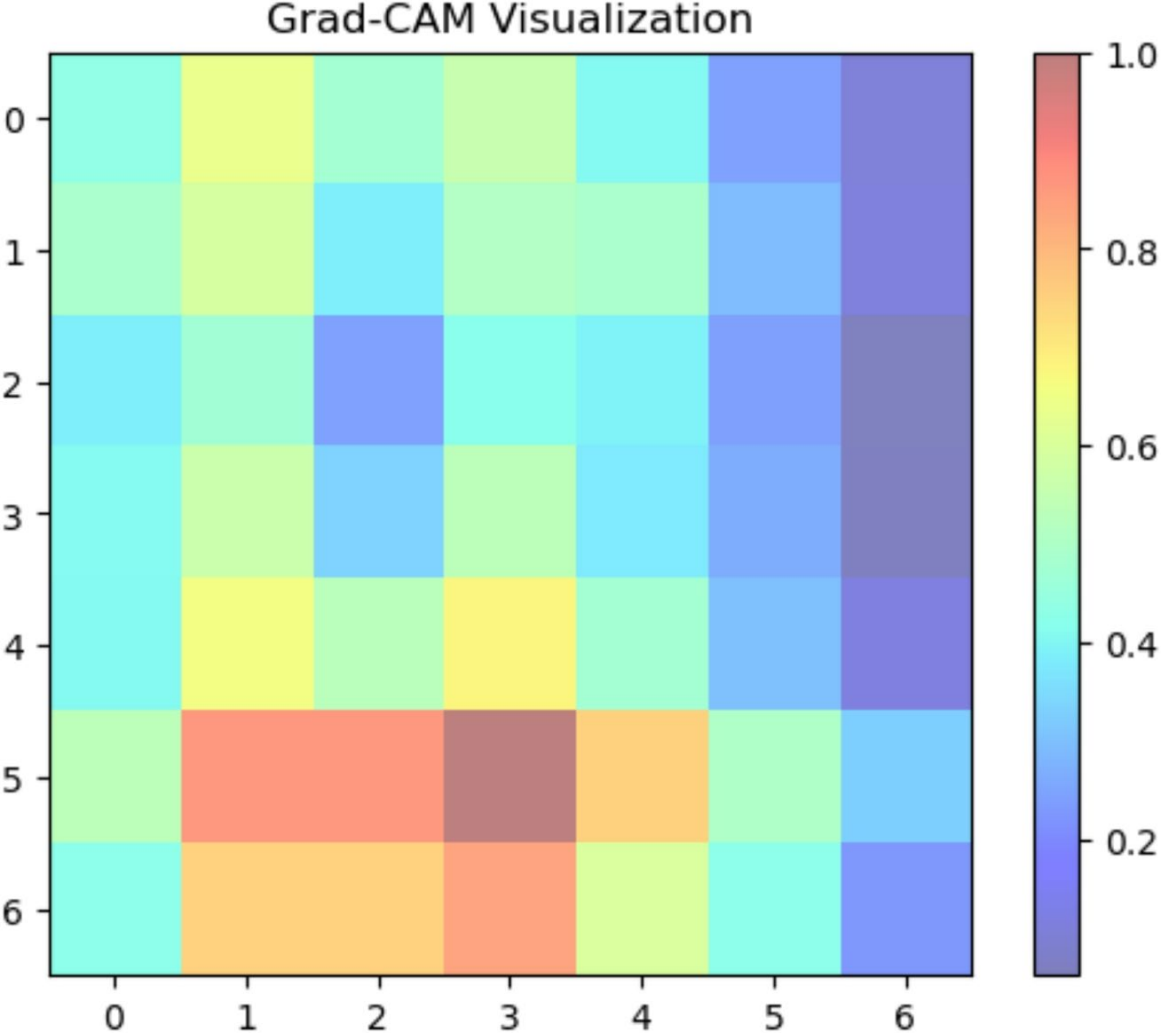
Grad-CAM activation map indicating mosaic pattern influence on classification.

**Fig. 10:**
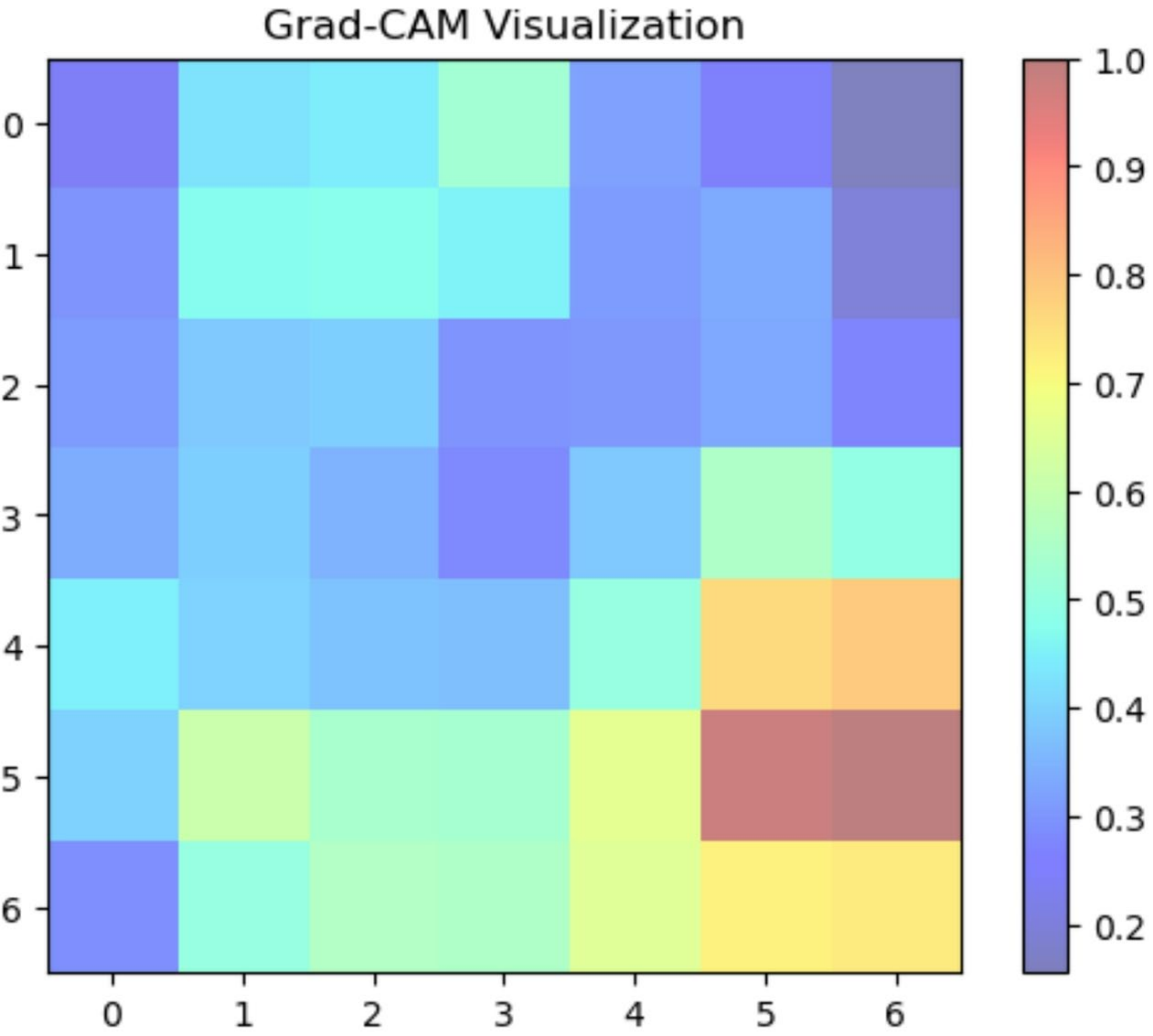
Grad-CAM activation map indicating yellow pattern influence on classification.

**Fig. 11:**
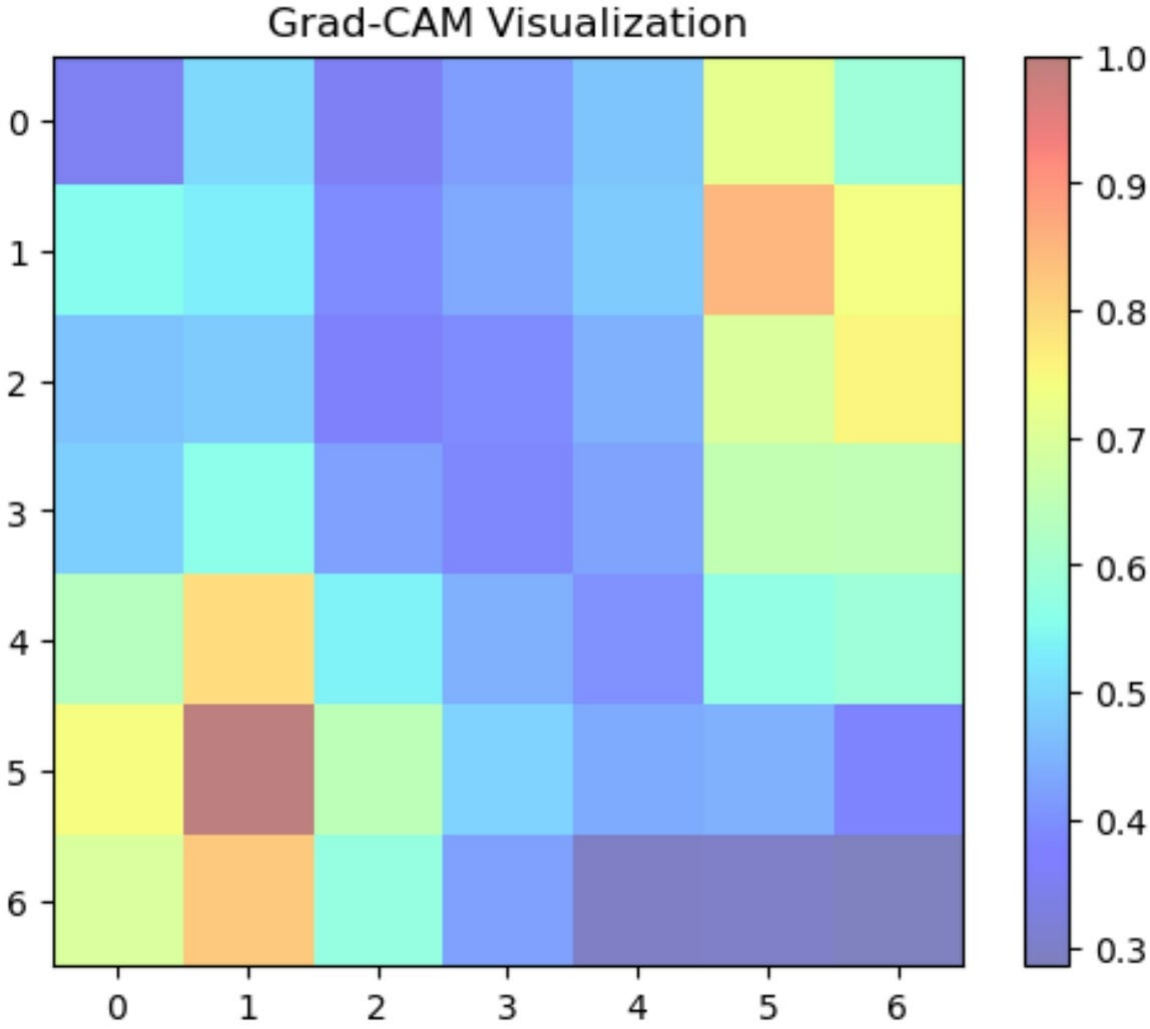
Grad-CAM activation map indicating ‘red rot pattern influence on classification.

**Fig. 12:**
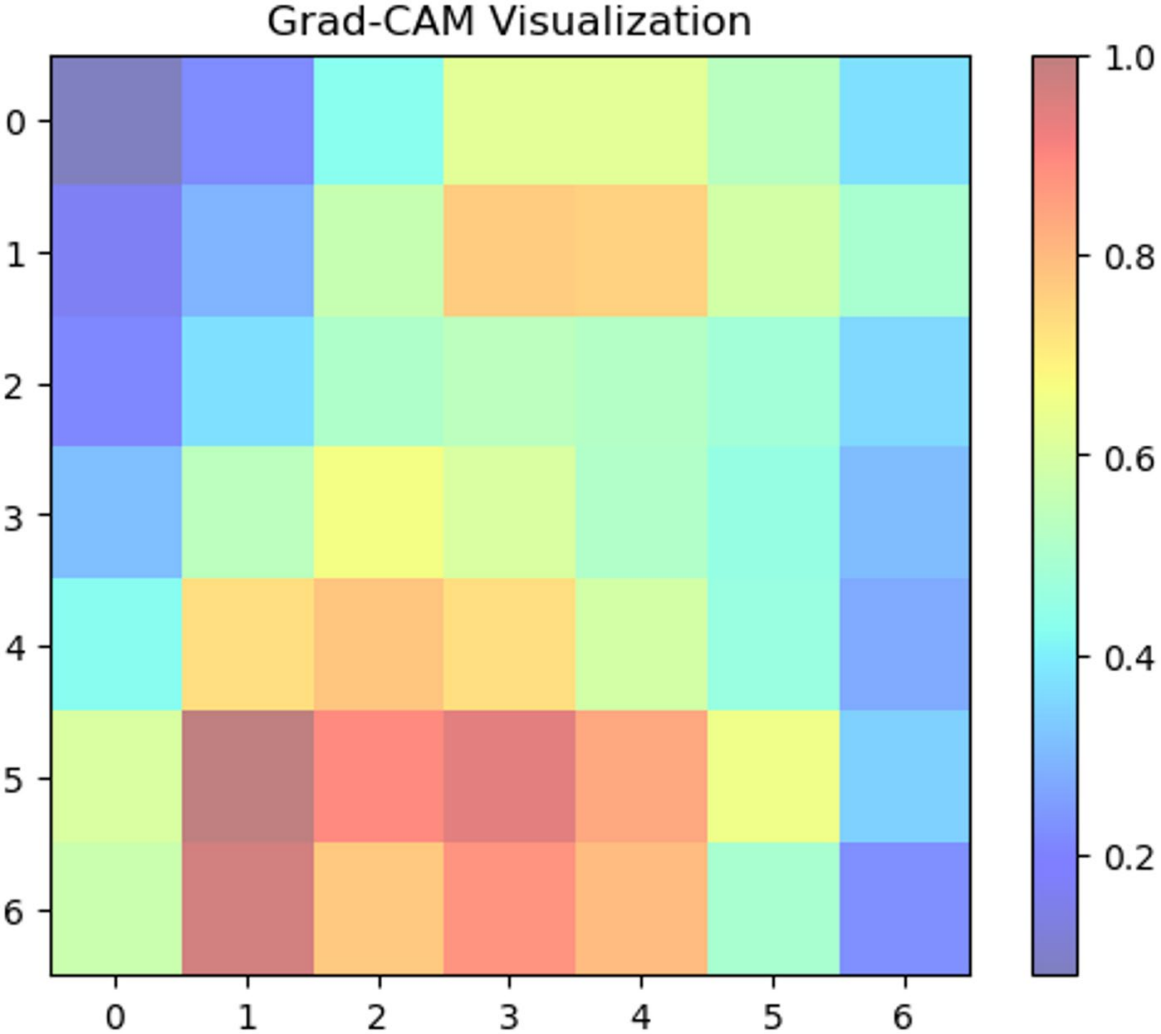
Grad-CAM activation map indicating rust pattern influence on classification.

We used Grad-CAM to create visual explanations for each disease category and healthy leaves to comprehend the factors influencing our deep learning model’s classification decisions (Fig. 8. The absence of certain localized defects was crucial for classification, as the attention maps for healthy leaves typically spanned the entire leaf area. On the other hand, the XAI identified areas linked to the recognized symptoms of each disease for the afflicted groups. For ‘Mosaic,’ the attention maps strongly highlighted the Fig. 9 leaf’s variegated discolouration patterns. In ‘Yellow Leaf Disease,’ the midrib and the yellowish patches that protruded from it were always emphasized, which matched the disease’s typical course in Fig. 10. For ‘Rust,’ the attention maps identified the tiny, rust-colored pustules on the leaf surface of Fig. 11, whereas for ‘Red Rot,’ the model concentrated on localized reddish or brownish lesions in Fig. 12. By showing that the model can link particular visual characteristics to every Sugar cane leaf disease, these XAI results offer insightful information about the model’s learning process and improve the predictability and interpretability of our model’s output. This knowledge can also guide the creation of more focused and effective disease detection techniques.

**Fig. 13:**
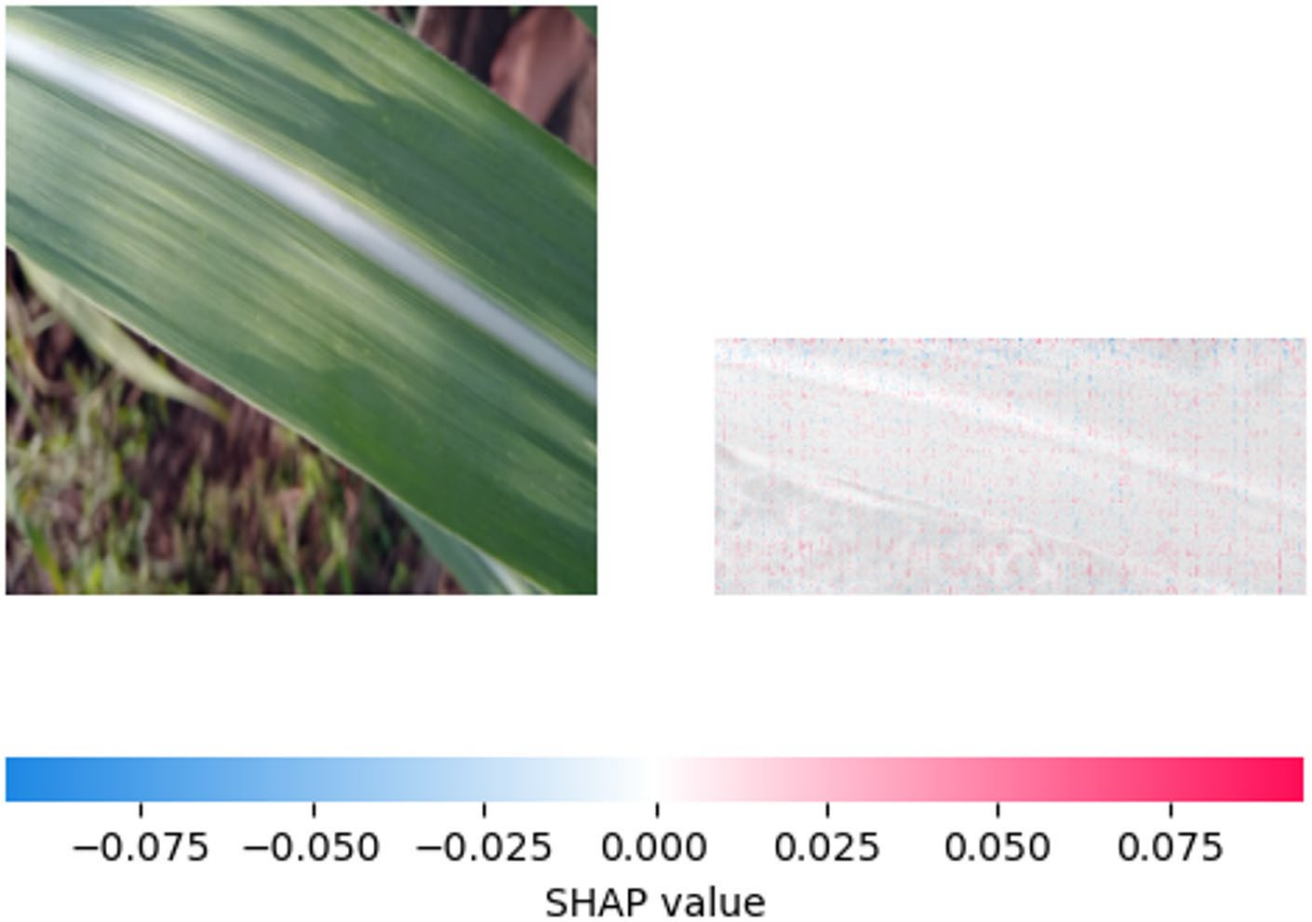
SHAP explanation indicating healthy pattern influence on classification.

Figure 13 shown feature importance analysis using SHAP values on a visually inspected leaf sample showed that the region exhibiting a lighter stripe had a low positive or even negative contribution to the ‘Healthy’ classification (SHAP value near zero or slightly negative ) .This indicates that the visual features in this discolored area do not strongly correlate with the characteristics the model identifies as indicative of healthy plant tissue.

**Fig. 14:**
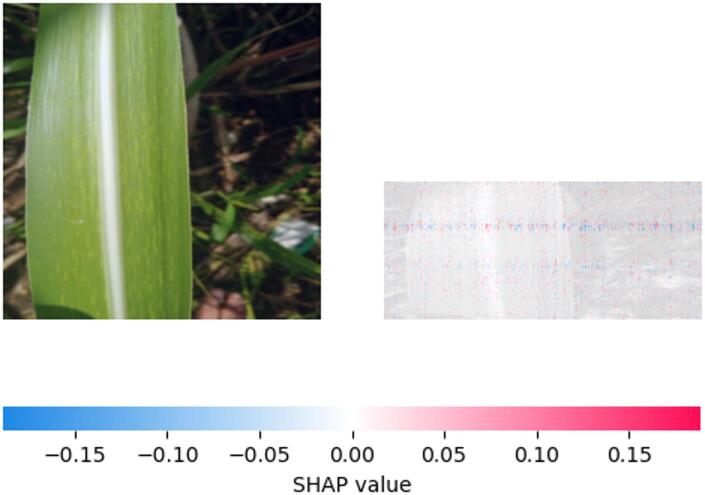
SHAP explanation indicating mosaic pattern influence on classification.

The relative significance of several visual signals for the model’s “Mosaic disease” prediction is shown by the SHAP map in Fig. 14. The elongated chlorotic stripe’s strong positive SHAP values suggest that this linear discoloration pattern is a crucial factor affecting the model’s conclusion. Lower or even negative SHAP values may be shown in other, less noticeable discolored patches, indicating a hierarchical significance of visual symptoms in the model’s diagnosis procedure.

**Fig. 15:**
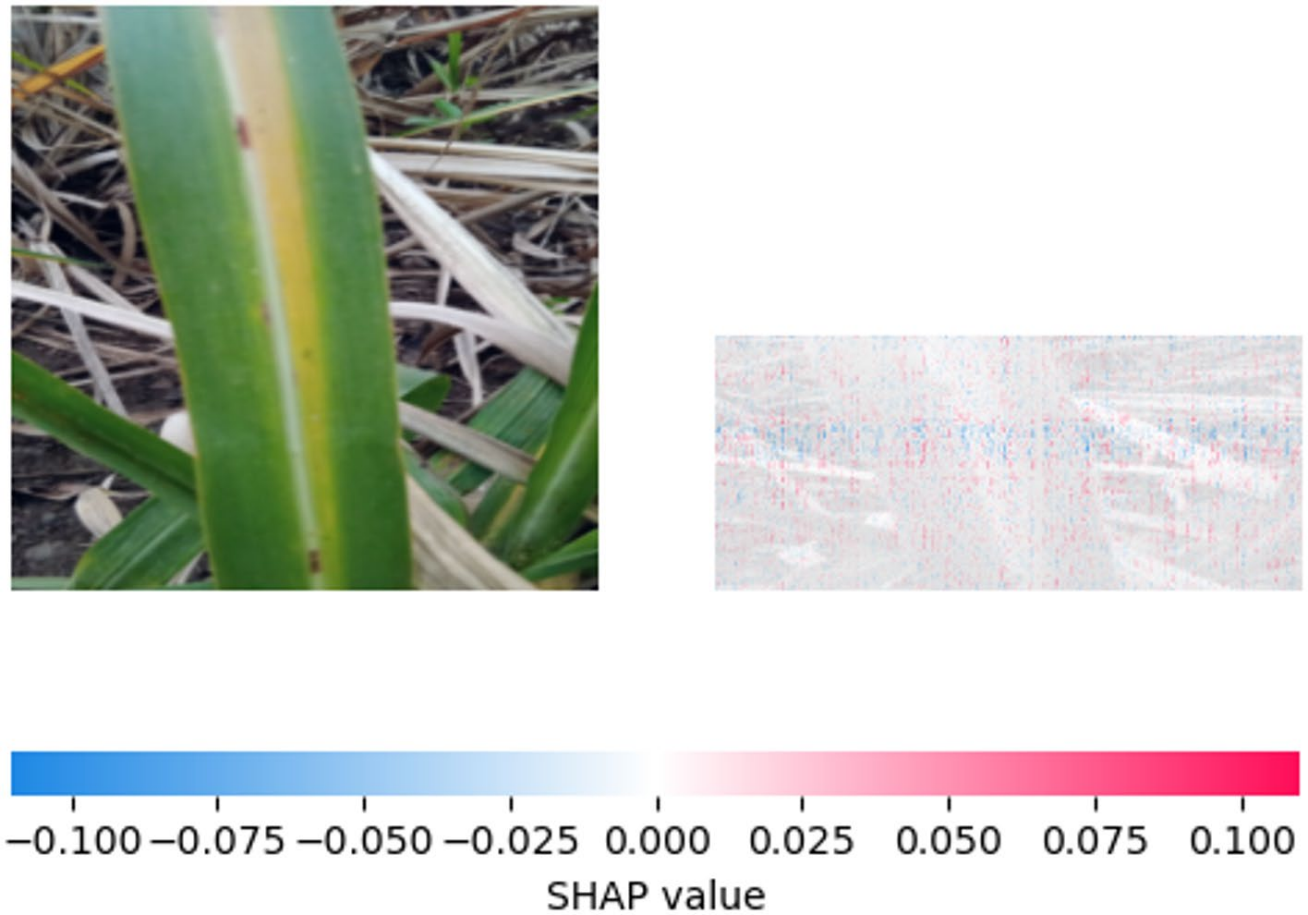
SHAP explanation indicating yellow pattern influence on classification.

Figure 15 illustrates the spatial contribution of different regions of the Sugar cane leaf to the model’s ‘Yellow disease’ prediction. The areas corresponding to the yellow stripe show the most positive SHAP values, emphasizing their significance in driving the classification towards ‘Yellow disease.’ However, in contrast to the negative values in the green areas, the magnitude of these positive values offers insight into the model’s confidence and the relative influence of healthy versus diseased tissue in the decision-making process.

**Fig. 16:**
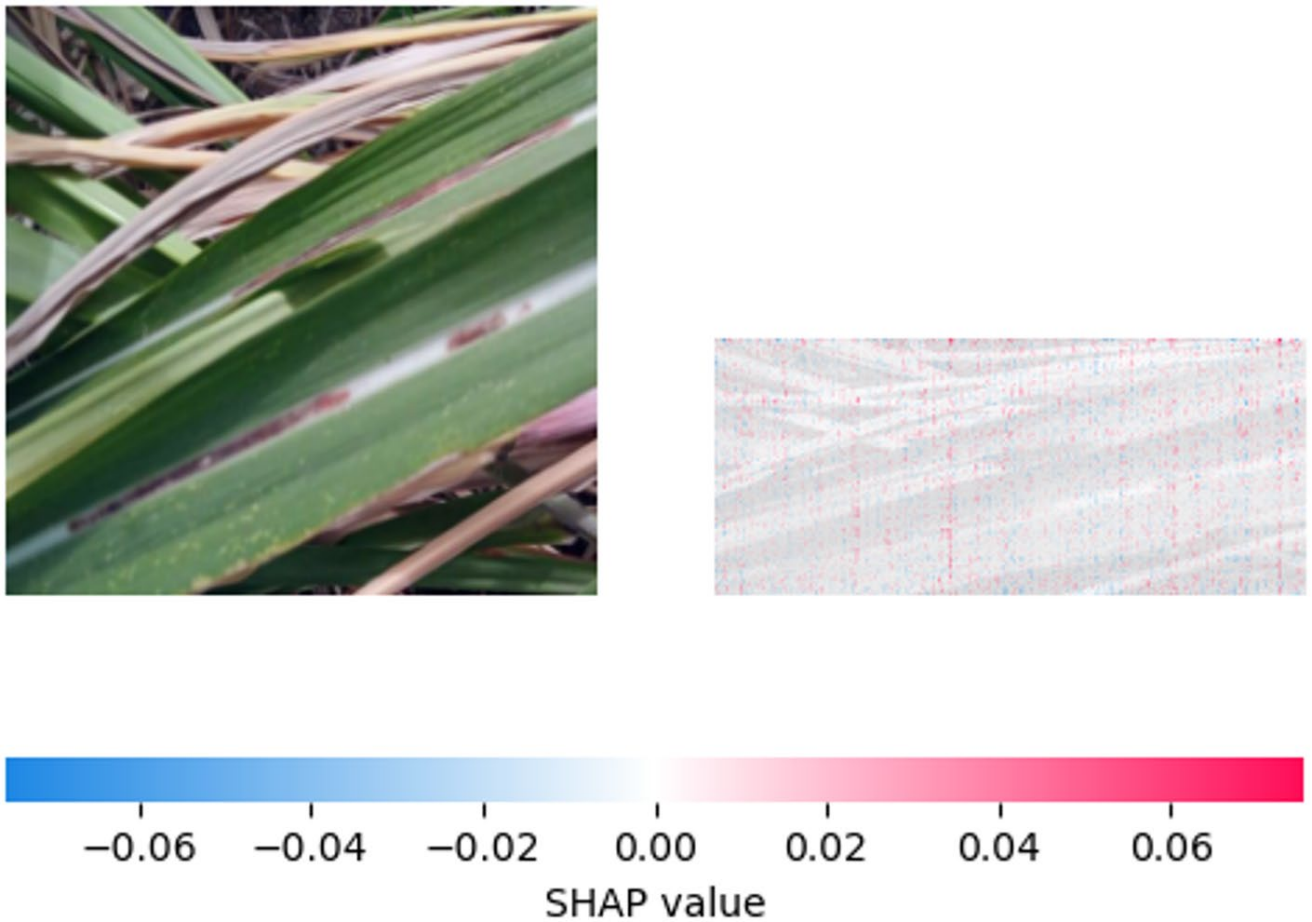
SHAP explanation indicating ‘red rot pattern influence on classification.

The relative significance of several visual characteristics on the Sugar cane leaves for the model’s “Redrot disease” prediction is shown by Fig. 16 .The regions exhibiting the highest positive SHAP values are those with pronounced reddish-brown tints and desiccation symptoms, indicating that these are important model indications. The distribution and size of these positive values throughout the afflicted leaves can be used to determine how sensitive the model is to different levels and patterns of Redrot symptoms.

**Fig. 17:**
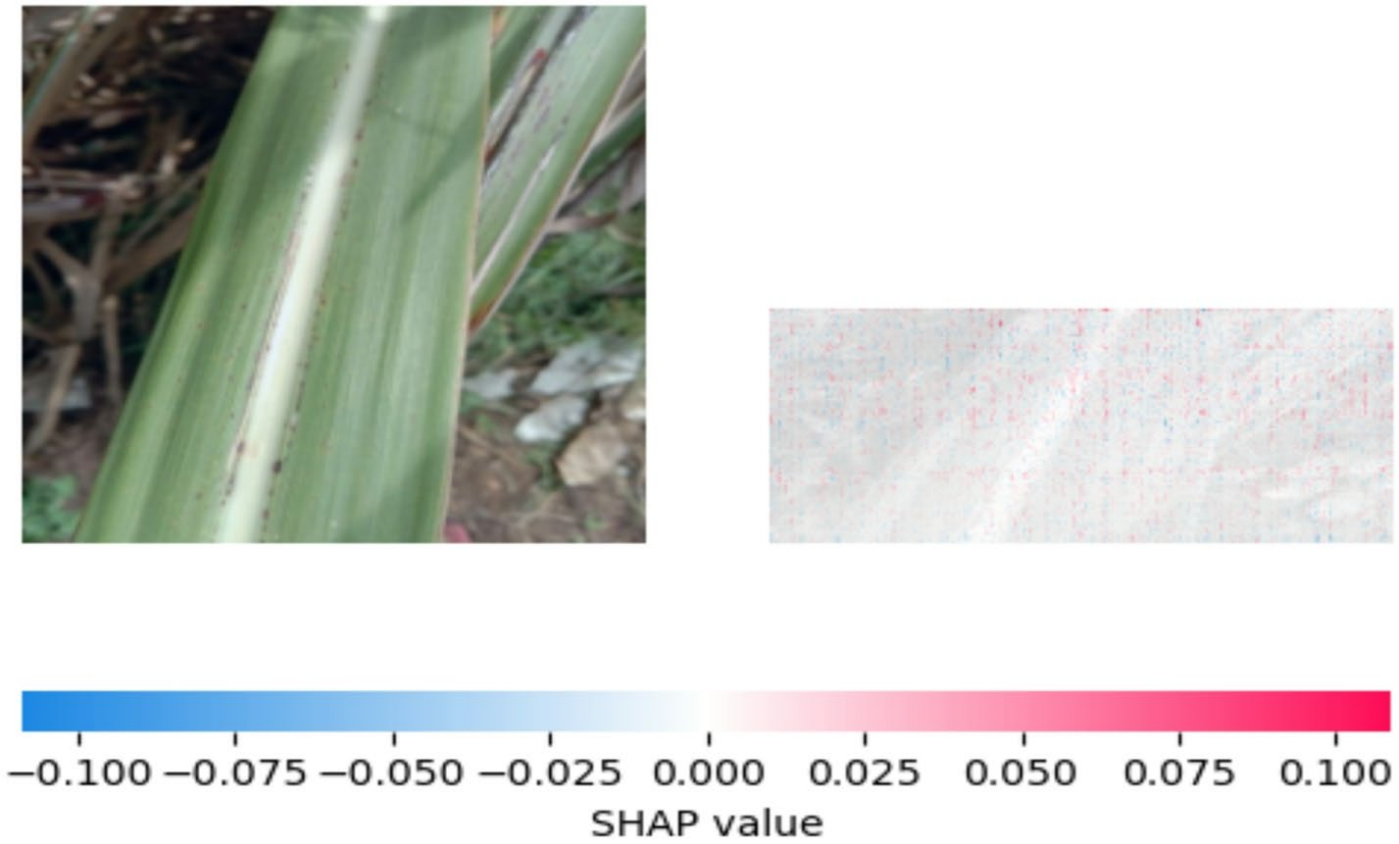
SHAP explanation indicating rust pattern influence on classification.

Areas on the Sugar cane leaves with reddish-brown pustules and streaks, which are typical signs of rust disease, correlate to regions with positive SHAP values, according to the SHAP analysis in Fig. 17. This suggests that the model has figured out how to link these visual characteristics to a greater risk of “Rust disease.” SHAP values are closer to zero or even slightly negative in the greener, ostensibly unaffected sections of the leaves, indicating that they contribute less to the positive illness classification. This discovery emphasizes how crucial these rust-specific lesions are to the automated identification of Sugar cane “Rust disease”.

### According to rice leaf dataset

**Fig. 18:**
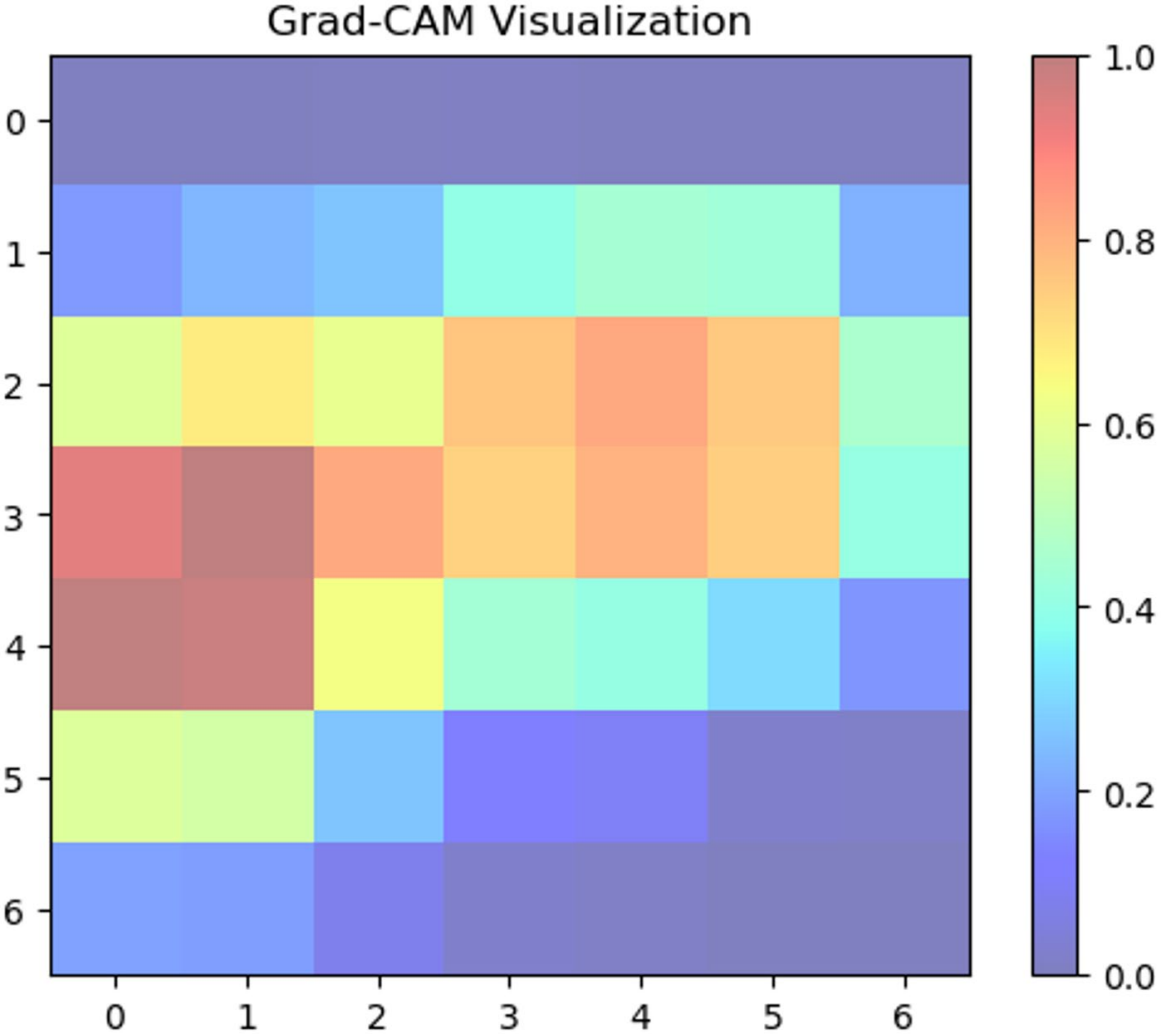
Grad-CAM activation map indicating bacterial leaf blight on classification.

Figure 18 shows the model’s forecast of Bacterial Leaf Blight is highly concentrated on leaf areas. The disease is characterized by irregular, frequently elongated, yellowish to brownish lesions, which correspond to the high activation zones. This implies that the CNN has mastered the ability to recognize and rank these essential visual characteristics for precise rice leaf bacterial leaf blight categorization. The algorithm appropriately assigns these regions less importance for the classification of disease, as evidenced by the reduced activation in the greener, ostensibly healthy areas.

**Fig. 19:**
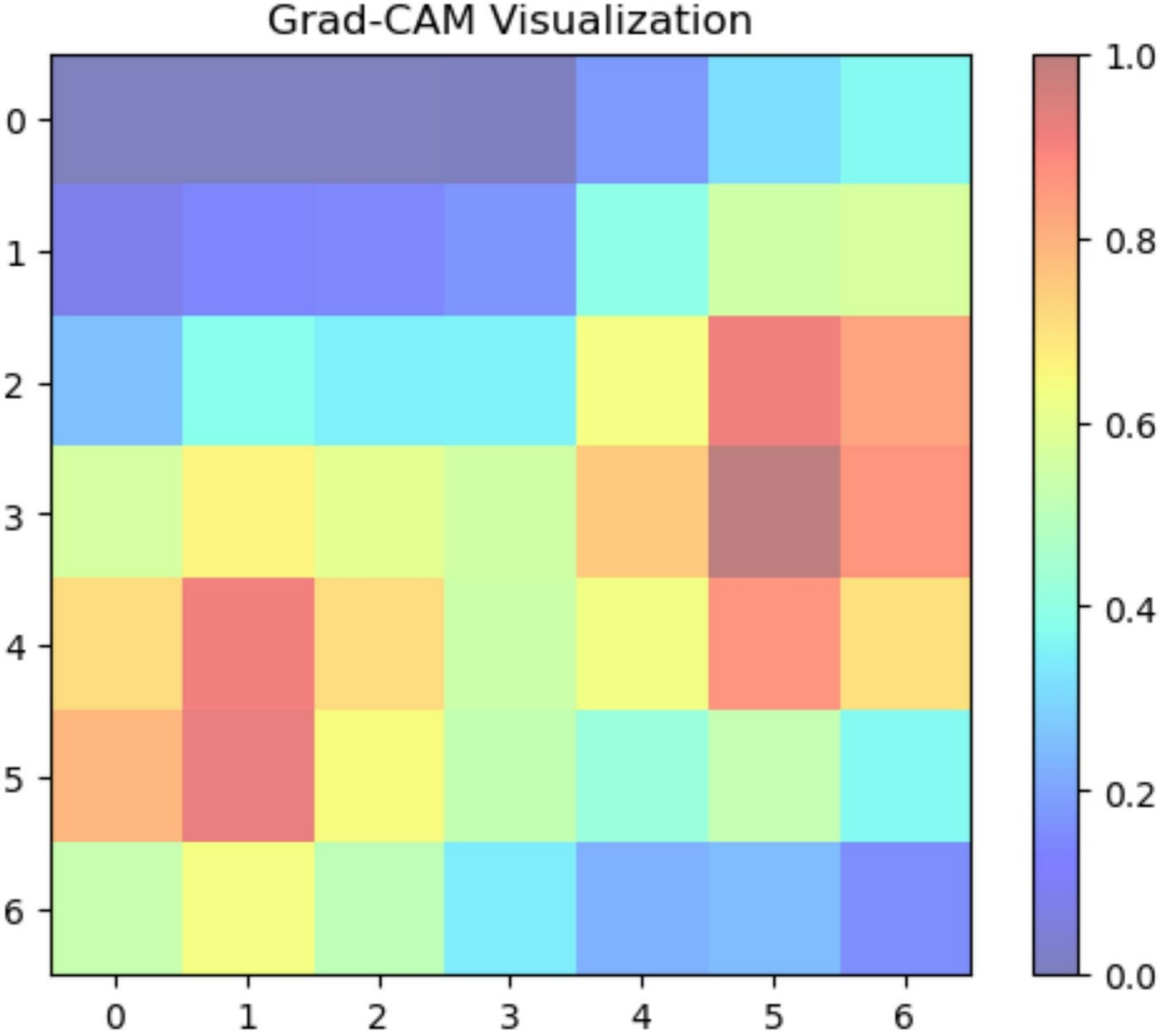
Grad-CAM activation map indicating brown spot on classification.

For the Brown spot categorization, Fig. 19 shows a rather diffuse pattern of high activation over a sizable section of the leaf. This implies that the model may be considering more general contextual information or more subtle alterations throughout the leaf surface rather than highly localized, disease-specific lesions. On the other hand, if the high activation areas appear in portions of the image that don’t seem to be related, it can mean that the model hasn’t mastered the ability to locate the Brown spot diagnostic features.

**Fig. 20:**
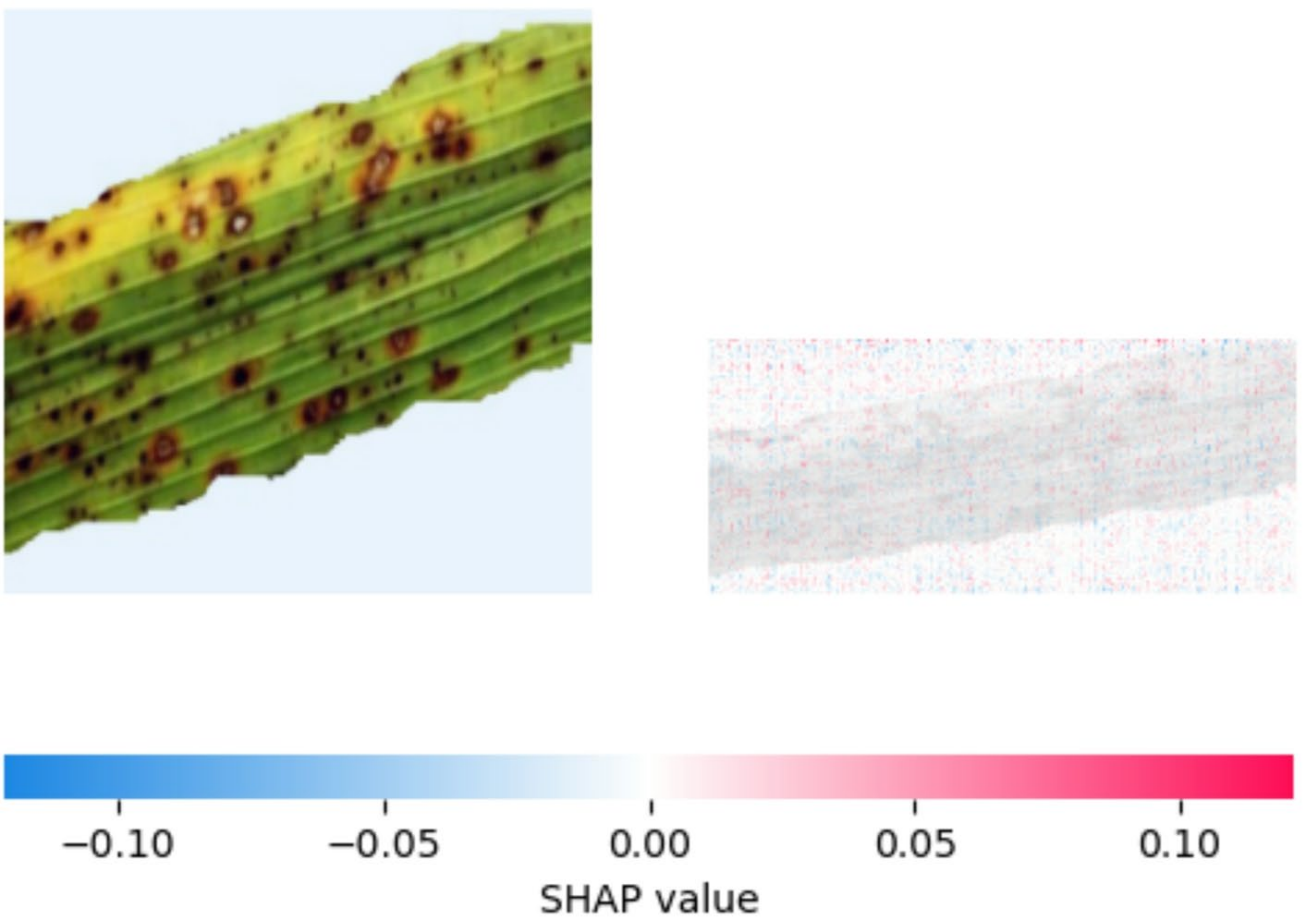
SHAP explanation indicating brown spot on classification.

Figure 20 shows that the areas of the leaf displaying the distinctive dark brown, frequently round or oval spots have a significant impact on the model’s prediction of Brown spot disease. Red pixels in the SHAP image indicate that the regions corresponding to these lesions have primarily positive SHAP values. This indicates that one of the main factors influencing the model’s classification as “brown spot” is the existence of these brown spots. Although less noticeable, the greener, apparently undamaged sections of the leaf typically have SHAP values that are closer to zero or even slightly negative (seen by the presence of blue pixels), indicating that they do not significantly contribute to the positive disease diagnosis. This result demonstrates that the model has successfully learned to link the disease categorization to the visual characteristics of Brown spot lesions.

**Fig. 21:**
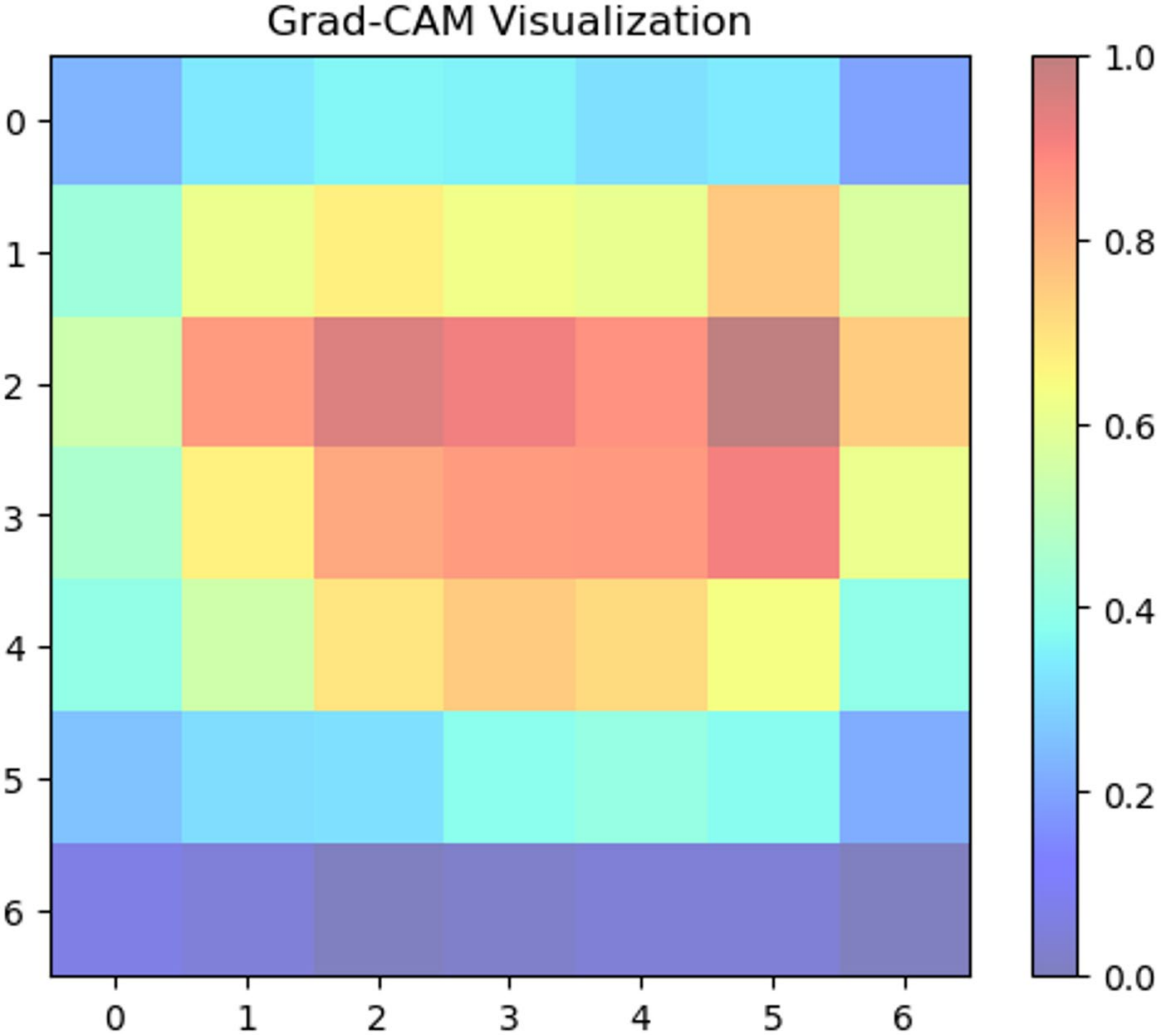
Grad-CAM Activation Map Indicating Leaf Smut on Classification.

A central, somewhat extended area of the leaf is the primary focus of the model’s classification of Leaf Smut illness, as shown in Fig. 21. The distinctive black, smutty streaks that appear along the leaf blade in this disease most likely match the high activation zones (seen by red and yellow hues). This implies that the CNN has mastered the ability to recognize and rank these essential visual characteristics for precise Leaf smut classification. The model’s capacity to distinguish between diseased and unaffected parts is further supported by the reduced activity in the surrounding, presumably healthier tissue.

**Fig. 22:**
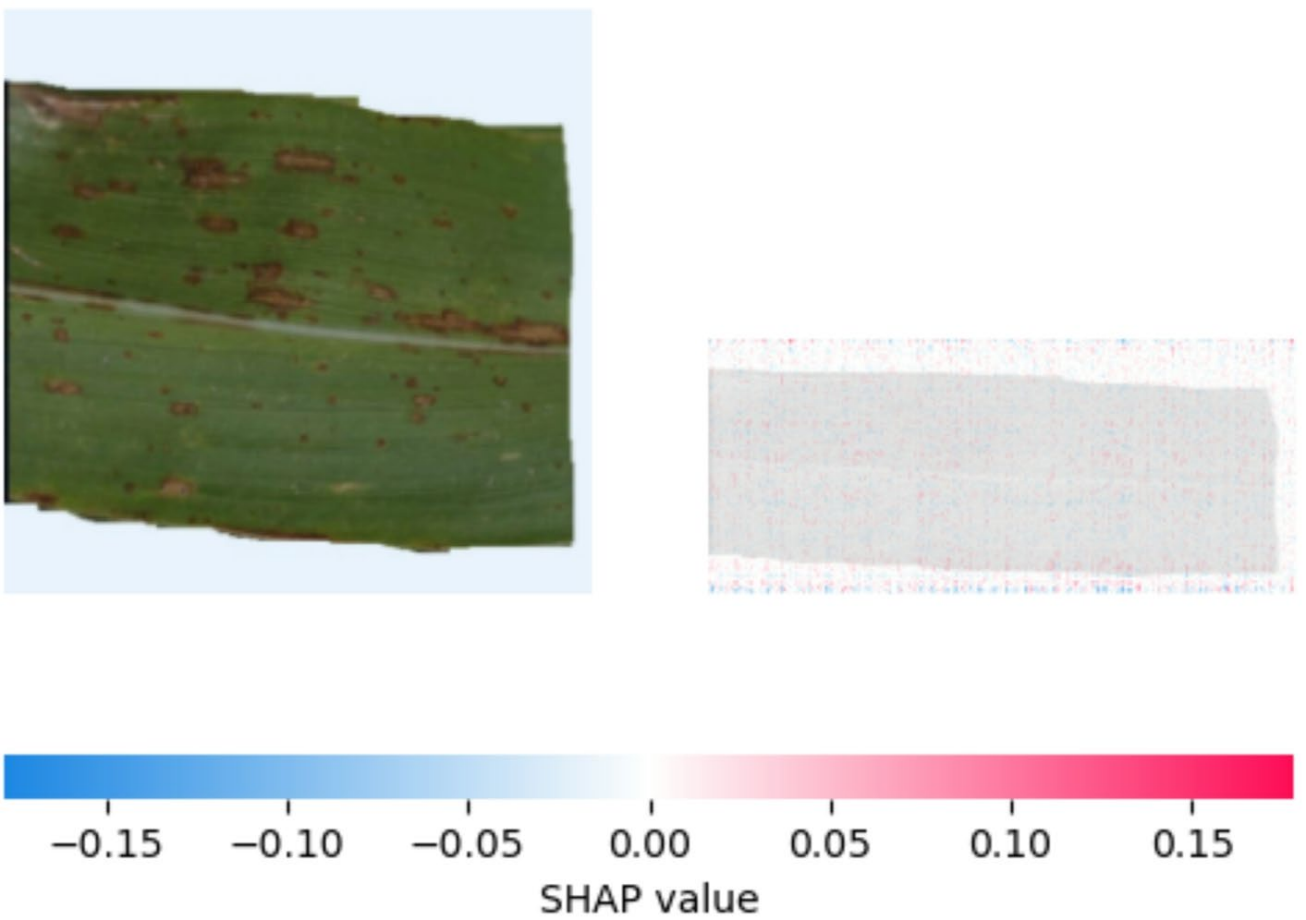
SHAP explanation indicating leaf smut on classification.

The presence of dark, elongated, smutty lesions dispersed throughout the leaf surface has a significant impact on the model’s prediction of Leaf Smut disease, as shown in Fig. 22. Red and pink pixels in the SHAP image show that the regions corresponding to these lesions have primarily positive SHAP values. This indicates that a greater probability of “Leaf smut” has been linked by the model to these distinctive dark streaks. On the other hand, as demonstrated by the presence of blue pixels, the greener, apparently unaffected sections of the leaf typically have SHAP values that are closer to zero or even slightly negative, indicating that they do not significantly contribute to the positive disease diagnosis. The model is concentrating on the pertinent visual cues for diagnosis, as evidenced by the dispersed pattern of positive SHAP values that closely matches the spread of the smut lesions seen in the leaf image.

## Conclusion and future scope

### Contributions summary

Using publicly accessible datasets, this work offers an explainable few-shot learning framework for rice and sugarcane leaf disease classification. The suggested system integrates CNN-based feature extraction, image preprocessing, data augmentation, and meta-learning methods like Prototypical Networks and MAML. The framework promotes trust and adoption in agricultural settings by providing transparent and interpretable disease predictions through the integration of XAI techniques such as Grad-CAM and SHAP.

### Practical implications

Explainable AI and few-shot learning work together to create a powerful decision-support tool for smart agriculture systems, particularly in situations where there is a lack of annotated data. Farmers and agronomists can improve field management, minimize crop losses, and aid in early diagnosis by highlighting disease-relevant regions and providing easily understood explanations.

### Future work

Better sensor–image combinations for early disease detection, field testing of the framework, and enhanced few-shot learning algorithms for greater robustness are all important areas for future research. Furthermore, creating XAI interfaces that are easier to use will encourage the widespread adoption and practical implementation of AI-driven agricultural solutions.

## Data Availability

No datasets were generated or analysed during the current study.
